# Draft Genomes of *Amaranthus tuberculatus*, *Amaranthus hybridus*, and *Amaranthus palmeri*

**DOI:** 10.1093/gbe/evaa177

**Published:** 2020-08-24

**Authors:** Jacob S Montgomery, Darci Giacomini, Bridgit Waithaka, Christa Lanz, Brent P Murphy, Ruth Campe, Jens Lerchl, Andreas Landes, Fanny Gatzmann, Antoine Janssen, Rudie Antonise, Eric Patterson, Detlef Weigel, Patrick J Tranel

**Affiliations:** e1 Department of Crop Sciences, University of Illinois, Urbana; e2 Max Planck Institute for Developmental Biology, Tübingen, Germany; e3 BASF SE, Limburgerhof, Germany; e4 BASF SE, Ludwigshafen, Germany; e5 Keygene N.V., Wageningen, The Netherlands; e6 Department of Plant, Soil and Microbial Sciences, Michigan State University

**Keywords:** *Amaranthus*, genome evolution, trio binning, chromatin contact mapping, linkage mapping, weed genomics

## Abstract

*Amaranthus tuberculatus*, *Amaranthus hybridus*, and *Amaranthus palmeri* are agronomically important weed species. Here, we present the most contiguous draft assemblies of these three species to date. We utilized a combination of Pacific Biosciences long-read sequencing and chromatin contact mapping information to assemble and order sequences of *A. palmeri* to near-chromosome-level resolution, with scaffold N50 of 20.1 Mb. To resolve the issues of heterozygosity and coassembly of alleles in diploid species, we adapted the trio binning approach to produce haplotype assemblies of *A. tuberculatus* and *A. hybridus*. This approach resulted in an improved assembly of *A. tuberculatus*, and the first genome assembly for *A. hybridus*, with contig N50s of 2.58 and 2.26 Mb, respectively. Species-specific transcriptomes and information from related species were used to predict transcripts within each assembly. Syntenic comparisons of these species and *Amaranthus hypochondriacus* identified sites of genomic rearrangement, including duplication and translocation, whereas genetic map construction within *A. tuberculatus* highlighted the need for further ordering of the *A. hybridus* and *A. tuberculatus* contigs. These multiple reference genomes will accelerate genomic studies in these species to further our understanding of weedy evolution within *Amaranthus*.

**Significance**

The production of reference genome assemblies has accelerated the study of basic biology and evolution within many species, but these resources have yet to be developed for many agronomically important weeds. This work reports new or improved genomes for three such weed species that will allow for the study of many biological questions.

## Introduction

The genus *Amaranthus* contains some of the most agronomically important weeds, including *Amaranthus tuberculatus* (Moq.) J.D. Sauer, *Amaranthus hybridus* L., and *Amaranthus palmeri* (S.) Watson (Sauer 1957, 1967). The ability of these *Amaranthus* species to evolve herbicide resistance via de novo mutation or interspecific hybridization makes them primary targets of weed management programs (Culpepper et al. 2006; Tranel et al. 2011; Gaines et al. 2012). The availability of high-quality reference genomes in *Amaranthus* species would allow for more robust genomic and molecular studies within these species including elucidating the evolution of weedy traits such as herbicide resistance, which are often the result of very strong selection pressures exerted across vast geographic distances and under many different environments (Korte and Farlow 2013; Patterson et al. 2019). These selection pressures afford a unique opportunity to explore evolution and ecology under extraordinary conditions. Furthermore, the creation of such resources would contribute to an international effort to develop tools for the study of genomics in the world’s worst weeds (Tranel and Trucco 2009; Ravet et al. 2018).

To date, some work on *Amaranthus* genomes has been published. Lightfoot et al. (2017) previously utilized Pacific Biosciences (PacBio) sequencing and Hi-C chromosome contact mapping to assemble and order the genome of *Amaranthus hypochondriacus*, an emerging pseudocereal crop species. Additionally, Kreiner et al. (2019) and Molin et al. (2020) reported the first genome assemblies of *A. tuberculatus* and *A. palmeri*, respectively.

In this work, we report the most contiguous draft assemblies of *A. tuberculatus*, *A. hybridus*, and *A. palmeri* to date. First, we produced haploid assemblies of *A. tuberculatus* and *A. hybridus* using the trio binning technique (Koren et al. 2018), which limited confounding effects from heterozygosity and avoided coassembly of alleles. This approach included generating PacBio sequences of an interspecific hybrid of *A. tuberculatus* and *A. hybridus*. Using short-read sequence data from each parent, the PacBio reads of this hybrid were binned based on short subsequences unique to each parental genome. The two resulting groups of PacBio reads were then assembled separately. Second, we assembled and ordered the *A. palmeri* genome into near-chromosome-level scaffolds using PacBio sequencing and Hi-C chromosome contact mapping. All assemblies were passed through repeat-masking and annotation pipelines. We conclude with a brief discussion of genome structure and synteny among *Amaranthus* species, obtained by comparing the newly produced genomes with the chromosomal organization of a closely related species, *A. hypochondriacus*. Synteny analysis included a version of the *A. tuberculatus* genome scaffolded based on recombination frequencies derived from segregating F_2_ populations, which identified chromosomal rearrangement not detected by anchoring the contigs to the pseudochromosomes of *A. hypochondriacus*.

## Materials and Methods

### Trio-Binned Genome Assemblies

#### Hybrid Development and Identification

An interspecific cross was made between a male *A. tuberculatus* plant of the ACR population, a population with dominant resistance to the herbicide imazethapyr (Patzoldt et al. 2005; Patzoldt and Tranel 2007), and an herbicide-sensitive *A. hybridus* plant. To identify interspecific hybrids, seed produced from this cross was scattered over moistened soil, treated with 1,066 g ae ha^−1^ of imazethapyr (Pursuit herbicide, BASF, Ludwigshafen, Germany), covered with a thin layer of soil, and watered regularly. Each survivor was tested with primer set MU_657.2 under conditions described by Montgomery et al. (2019) to confirm hybrid identity and identify male hybrids.

#### Parental Sequencing

Genomic DNA was extracted from the two parents of the cross between *A. tuberculatus* and *A. hybridus* according to a described protocol (Xin and Chen 2012) and used to generate short-read sequencing libraries. These libraries were then sequenced to a coverage of ∼100× on a HISEQ 3000 instrument **(**Illumina, San Diego, CA) using a HISEQ3000/4000 SBS kit and a paired-end 150 base read chemistry.

#### Hybrid Sequencing

High-molecular weight genomic DNA was extracted from an identified hybrid and used to create SMRTbell templates, which were sequenced on a Sequel II system (Pacific Biosciences, Menlo Park, CA).

#### Haplotype Assembly and Polishing

About 116.8 Gb of Sequel II long-read data were trio binned using the trio bin feature of *Canu*, and the two haplotypes were subsequently assembled using *Canu* (version 1.8; correctedErrorRate=0.04 corMhapSensitivity=normal genomeSize=XXX corMhapFilterThreshold=0.0000000002 corMhapOptions=“--threshold 0.80 --num-hashes 512 --num-min-matches 3 --ordered-sketch-size 1,000 --ordered-kmer-size 14 --min-olap-length 2000 --repeat-idf-scale 50” mhapBlockSize = 500 ovlMerDistinct = 0.975; Koren et al. 2017). The parameter genomeSize was set to 700 and 500 m for assembly of *A. tuberculatus* and *A. hybridus*, respectively. The binned subreads from each species were aligned to their respective assemblies using *Minimap2* (version 2.17; --sort, other parameters default; Li 2018). This alignment was used to polish the raw contigs with *Arrow* (*pbgcpp*, version 1.9.0). Both assemblies were also finished against the *A. hypochondriacus* genome (Lightfoot et al. 2017) using *REVEAL finish* (commit 98d3ad1; Kreiner et al. 2019). Depth of coverage was calculated using *samtools depth* and *Minimap2* alignments (version 1.7; Li et al. 2009), and plotted in R (R Core Team 2018).

#### Haplotype Assembly Annotation

Parallel methodologies were used to repeat-mask and annotate the *A. tuberculatus* and *A. hybridus* genomes in *GenSaS* (version 6.0; Humann et al. 2019). In each species, a library of predicted repeats, generated with *RepeatModeler* (version 1.0.11; www.repeatmasker.org), was combined with a library of repeats identified using *RepeatMasker* (version 4.0.7; Smit et al. 2013) and an *Arabidopsis thaliana* repeat library within *GenSaS* to create a consensus library of repeats and mask each polished assembly. Nucleotide and protein sequence of *A. tuberculatus*, *A. palmeri*, *A. hypochondriacus* reference transcriptomes (Riggins et al. 2010; Giacomini D, unpublished data; Lightfoot et al. 2017) were aligned to each assembly using BlastN (version 2.7.1; Camacho et al. 2009) and BlastX (version 2.6.0; Camacho et al. 2009), respectively. These alignments were combined with results of gene prediction modeling (*AUGUSTUS*; version 3.1.1; Stanke et al. 2004) to generate an official gene set and identify predicted transcripts within each masked assembly (EVidenceModeler, release June 25, 2012, Haas et al. 2008). Predicted transcripts were prescribed function based on alignment (BlastP; version 2.2.28, -evalue 1e-6 -max_hsps_per_subject 1 -max_target_seqs 1; Camacho et al. 2009) to the UniProtKB database.

#### Genetic Map Construction of A. tuberculatus

Two pseudo-F_2_ mapping *A. tuberculatus* populations, POP1 and POP2, were established from single plant crosses, in which the same male *A. tuberculatus* plant was used for both crosses. Whole-genome sequence was generated for each parent using a Hi-Seq 4000 instrument, yielding 150-bp paired-end reads. Double-digest restriction-site associated DNA sequencing libraries were generated following Montgomery et al. (2019) for 285 individuals randomly selected from each population. Libraries were sequenced using a NovaSeq S1 flowcell (Illumina, San Diego, CA). Variant calling was conducted following the GATK 4.0 pipeline following best practices recommendations with variants hard filtered (FS>20, MQ<50, MQRankSum<−2, −3 < ReadPosRank Sum<4, SOR>4, QD<10; Poplin et al. 2017). The filtering pipeline *filter_variants_mapping.sh* was implemented to obtain mapping quality variants and is available through GitHub (brentpm2/genetic_map_tuberculatus). Retained variants were observed in parent and at least 10% of pseudo-F_2_ individuals. Contigs with two or more variants were retained within 100 individuals per population, and missing data imputed with *Beagle* (version 4.0, Browning and Browning 2007). Variants that deviated from the expected 1:2:1 segregation ratio were removed. A genetic map was constructed with R/qtl independently for each population, where marker order on each contig was retained during analysis, and contig order was compared (Broman et al. 2003).

### *Amaranthus palmeri* Genome Assembly

Several *A. palmeri* populations were passed through a previously described sequence-based genotyping pipeline (Truong et al. 2012) to quantify the level of heterogeneity and heterozygosity present within each population and plant, respectively. The population LIH06329 showed a dense cluster in a principal component analysis formed by individual plants, indicating low heterogeneity. Additionally, plants from this population showed sensitivity to multiple herbicides tested from eight herbicide modes-of-action classes 2, 4, 5, 6, 9, 10, 14, and 27 (see https://hracglobal.com/tools/hrac-mode-of-action-classification-2020-map; last accessed September 2, 2020). The 30 representative plants from LIH06329 had an average heterozygosity measure of 26.8% based on sequence information at 5556 loci generated through the sequence-based genotyping pipeline mentioned above. Leaf material from the plant with the lowest heterozygosity measure (21.2%) was harvested and used for sequencing.

Part of this material was sent to Dovetail (Scotts Valley, CA) to prepare and sequence Hi-C (211 M read pairs; 150 bp) and Chicago (235 M read pairs; 150 bp) libraries for chromosome scale scaffolding. High-molecular weight genomic DNA was extracted from the remaining leaf material. This DNA was used to generate ∼20-kb insert size selected PacBio Sequel I libraries, which were sequenced using DNA 2.1 Polymerase chemistry.

About 106 Gb of long-read data were used to produce a de novo genome assembly using HGAP 5.1 (Chin et al. 2013). Raw reads were used to polish the resulting contigs as described above using *Arrow* (SMRTlink version 5.1.0). The polished assembly was provided to Dovetail to perform chromosome scale scaffolding based on chromatin contact mapping data generated through a Hi-C approach using the HiRise method (Putnam et al. 2016) and further improved by gap-filling using PBJelly (English et al. 2012). Regions of high heterozygosity that were falsely separated in this haploid assembly were purged using Haplomerger2 (filter_score2 = 500k, minOverlap = 10k; Huang et al. 2017). These scaffolds were passed through a repeat-masking and annotation pipeline analogous to the one described above for *A. tuberculatus* and *A. hybridus*.

### Interspecific Synteny Comparison

To identify differences in genomic structure and detect regions of synteny between the species described above, *A. palmeri* scaffolds, polished contigs of *A. tuberculatus* and *A. hybridus*, and *A. tuberculatus* scaffolds produced using the genetic map were each compared with the 16 pseudochromosomes of the *A. hypochondriacus* genome (Lightfoot et al. 2017) using Synmap2 (Legacy Version=True, Syntenic Path Assembly=True; Haug-Baltzell et al. 2017). The genome of *A. hypochondriacus* was also compared with itself to visualize endogenous duplications across the genome.

## Results and Discussion

The trio binning technique successfully isolated sequence from each haplotype of the *A. tuberculatus*×*A. hybridus* hybrid. Of 116.8-Gb PacBio subread sequence produced, 35.3% was binned as *A. hybridus*, 64.4% as *A. tuberculatus*, and only 0.1% as ambiguous, with the remaining 0.2% removed because reads containing this sequence were shorter than 1 kb. The higher proportion of sequences belonging to the *A. tuberculatus* haplotype is partially explained by differences in genome size: haploid genome sizes previously were estimated to be 675 and 503 Mb for *A. tuberculatus* and *A. hybridus*, respectively (Stetter and Schmid 2017).

Independent assembly of each bin resulted in the first reported *A. hybridus* reference genome assembly and a more contiguous assembly than the previously reported *A. tuberculatus* reference (Kreiner et al. 2019; table 1). Discrepancies between estimated and assembled genome size in these two species are likely due to the lack of centromeric and telomeric regions in our assemblies (Lamb and Birchler 2003; Kim et al. 2019). Additionally, because only one haplotype was included in the assembly process, we are confident that multiple alleles do not inflate our assembly size. This conclusion is supported by the presence of only one peak in the distributions of coverage across these assemblies, circumventing the need for additional finishing steps, such as haplotype merging (supplementary fig. S1, Supplementary Material online). By anchoring each of these genomes against the reference *A. hypochondriacus* assembly, we placed 99.6% of the *A. tuberculatus* and 99.8% of the *A. hybridus* assemblies onto the 16 pseudochromosomes of the *A. hypochondriacus* assembly. Our assembly of a male *A. tuberculatus* individual complements the previous assembly of a female (Kreiner et al. 2019), allowing for future comparative studies that may elucidate the genetic basis of dioecy in this species. The success of trio binning to produce *A. tuberculatus* and *A. hybridus* genome assemblies sets a precedent for this technique to be used in other plant species that produce viable interspecific hybrid progeny, such as *Ambrosia trifida* and *Ambrosia artemisiifolia* (Vincent and Cappadocia 1987).

**Table 1:** Summary Statistics of *Amaranthus palmeri*, *Amaranthus tuberculatus*, and *Amaranthus hybridus* Draft Genome Assemblies

	*A. palmeri*	*A. tuberculatus*	*A. tuberculatus* (Kreiner et al. 2019)	*A. hybridus*
Estimated genome size (*n*, Mb)^a^	421.8	675.6	675.6	503.8
Assembly size (Mb)	408.1	572.9	663.7	403.0
Number of contigs	638	841	2,514	640
Number of scaffolds	303	16^b^	16^b^	16^b^
Contig N50 length (Mb)	2.54	2.58	1.74	2.26
Scaffold N50 length (Mb)	20.11	34.7^b^	43.1^b^	24.5^b^
Annotated genes	29,758	26,784	56,936	24,325
Complete BUSCO	84.2%	81.9%	88%	89.8%

a Genome size estimates from cytological studies by Stetter and Schmid (2017).

b Scaffolded with respect to *Amaranthus hypochondriacus* pseudochromosomes (Lightfoot et al. 2017).

The linkage maps generated for POP1 and POP2 from *A. tuberculatus* agreed in contig order (supplementary table S1 and fig. S2, Supplementary Material online). As a shared parent is observed in each population, this suggests the lax variant filtration was still reflective of the overall genome structure. The map of POP1 was more complete than POP2, representing 74% of the total genomic sequence across 16 linkage groups. Although not sufficient for the complete scaffolding of the *A. tuberculatus* genome, the map allowed for a better comparison of synteny between *A. tuberculatus* and *A. hypochondriacus* (discussed below).

Our genome assembly pipeline for *A. palmeri* resulted in a highly contiguous reference genome with some chromosomes seemingly being assembled from end-to-end (table 1 andfig. 1). Recombination frequency across the genome could be derived from future linkage studies to validate the order of these contigs and further develop this genomic resource. These results confirm the efficacy of this “PacBio-plus-Hi-C” genome assembly approach, even in species known to be highly heterozygous (Laforest et al. 2020).

**Fig. 1.**
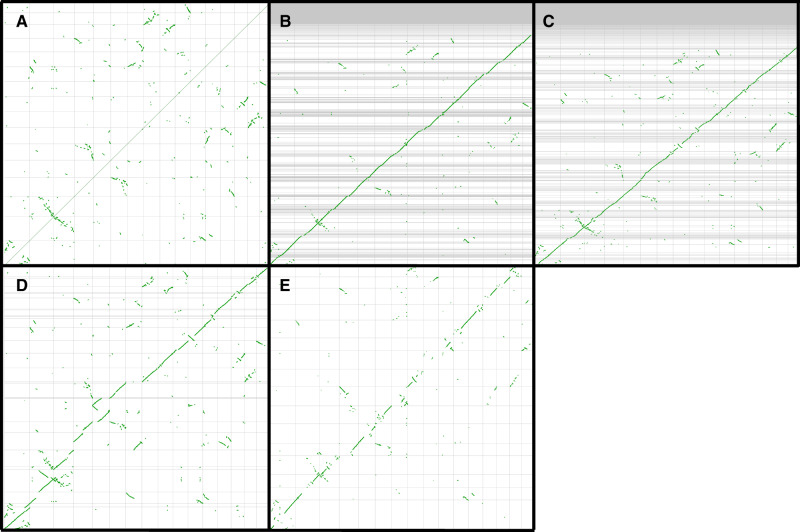
Dot plots demonstrating synteny between the genome of *Amaranthus hypochondriacus* and itself (*A*), the contigs of *Amaranthus hybridus* (*B*), and *Amaranthus tuberculatus* (*C*) as well as the scaffolds of *Amaranthus palmeri* (*D*). Panel (*E*) is an analogous plot comparing the *A. tuberculatus* contigs arranged according to linkage map generated from POP1 and the scaffolds of *A. hypochondriacus*. Within each panel, the 16 pseudochromosomes of *A. hypochondriacus* are represented along the *X* axis, separated by vertical lines. The contigs/scaffolds of genome assemblies from each other species are represented along the *Y* axis of their respective panes, separated by horizontal lines. Each green dot represents one syntenic gene, and obvious diagonals indicate syntenic regions between the two compared species.

To identify structural differences in each species’ genome, syntenic comparisons between each assembly and the 16 pseudochromosomes of *A. hypochondriacus* were made with Synmap2 (fig. 1). No major differences in chromosome structure were identified between *A. hypochondriacus* and *A. hybridus*, which was expected, as recent phylogenetic studies suggest these two species to be very closely related (Waselkov et al. 2018). Several small-scale inverted regions were detected within the contigs of the *A. tuberculatus* assembly. It is important to recognize, however, that without a reliable method of ordering the contigs, it was not possible to identify larger scale rearrangement events. In fact, ordering of *A. tuberculatus* contigs based on linkage mapping revealed additional rearrangements (fig. 1). Because synteny does not appear perfectly conserved within the *Amaranthus* genus, alternative scaffolding approaches should be implemented to finish the *A. tuberculatus* genome. Conversely, with the contiguity offered via chromatin contact mapping data obtained for *A. palmeri*, several large-scale inverted and translocated regions were seen relative to *A. hypochondriacus*. Additionally, the fragmentation of the chromosome 7 homolog in *A. palmeri* might be attributed to the previous observation that *A. palmeri* contains an additional pair of chromosomes compared with the other *Amaranthus* species included in this study (Grant 1959). Ultimately, these genomic resources will serve as valuable tools for genomic studies within these species to understand an array of biological questions, with their weediness attributes of particular interest.

## Supplementary Material

Supplementary data are available at *Genome Biology and Evolution* online.

## Supplementary Material

evaa177_Supplementary_Data

## Notes

**Data deposition:** This project has been deposited at CoGe at https://genomevolution.org/coge/ under accessions 56882, 56678, 57896, 57427, 57429, and 55760; and with the National Center for Biotechnology Information under accessions PRJNA641102, PRJNA640356, and PRJNA655730.
