# Supplementary material for: Draft Genomes of Amaranthus tuberculatus, Amaranthus hybridus, and Amaranthus palmeri
Source: Genome Biol Evol. 2020 Aug 24;12(11):1988–93. doi: 10.1093/gbe/evaa177 (PMC7643611; doi:10.1093/gbe/evaa177)
Supplement: evaa177_Supplementary_Data [file evaa177_supplementary_data.zip › Figure S2.pdf]

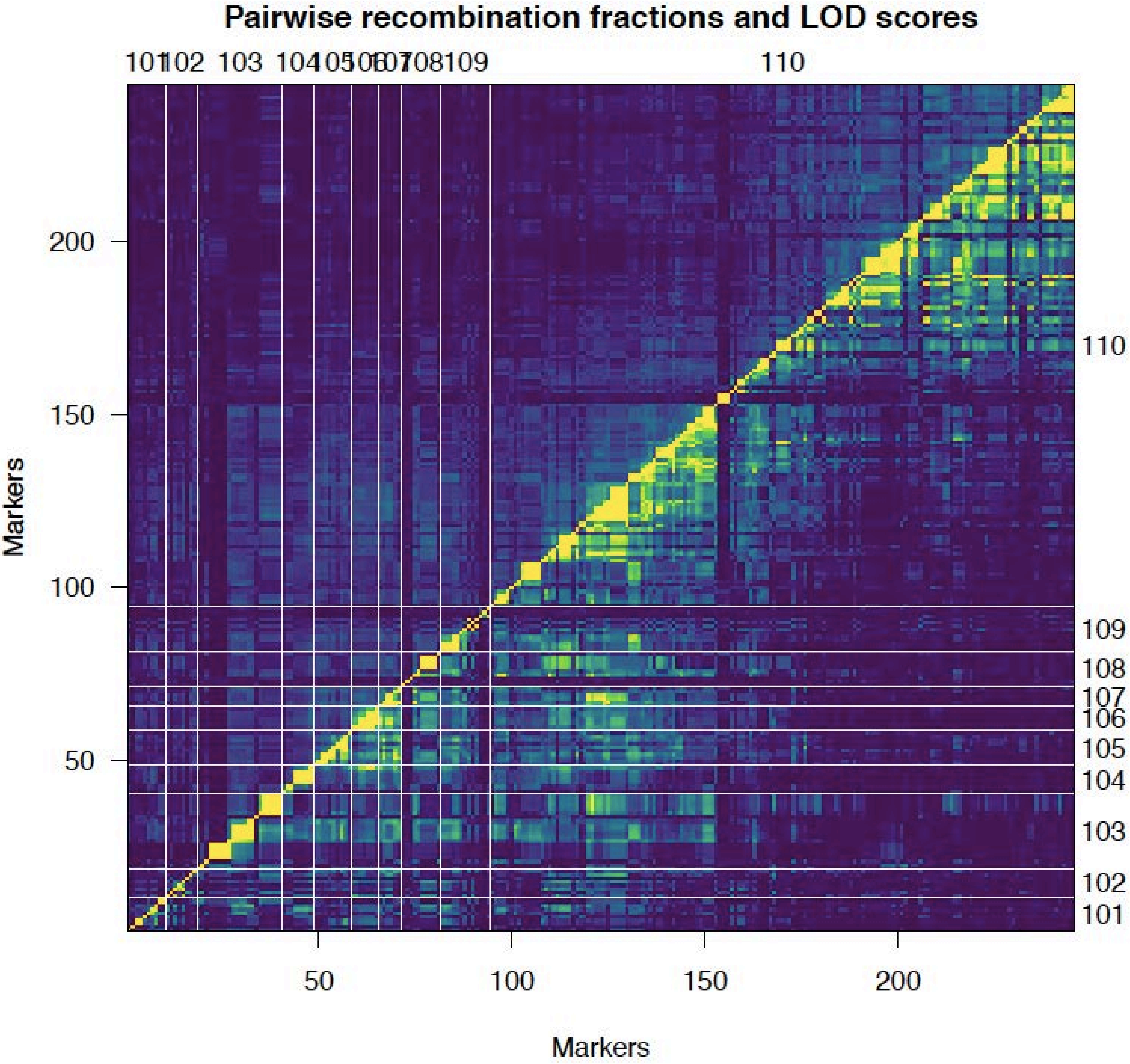

Pairwise recombination fractions and LOD scores
101
102
103
104
105
106
107
108
109
110
Markers
50
100
150
200

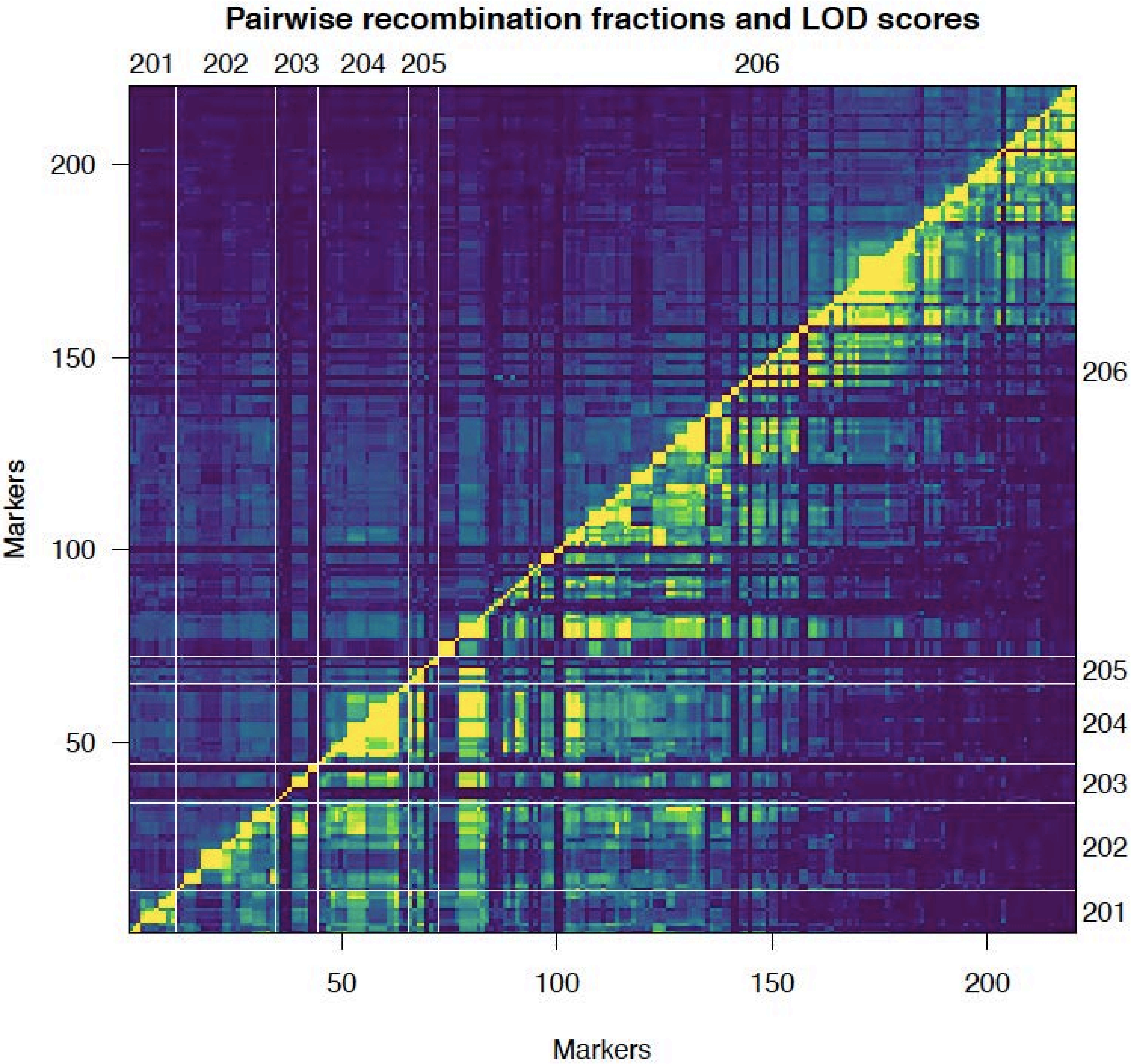

Pairwise recombination fractions and LOD scores
201
202
203
204
205
206
200
150
100
50
Markers
206
205
204
203
202
201
50
100
150
200
Markers

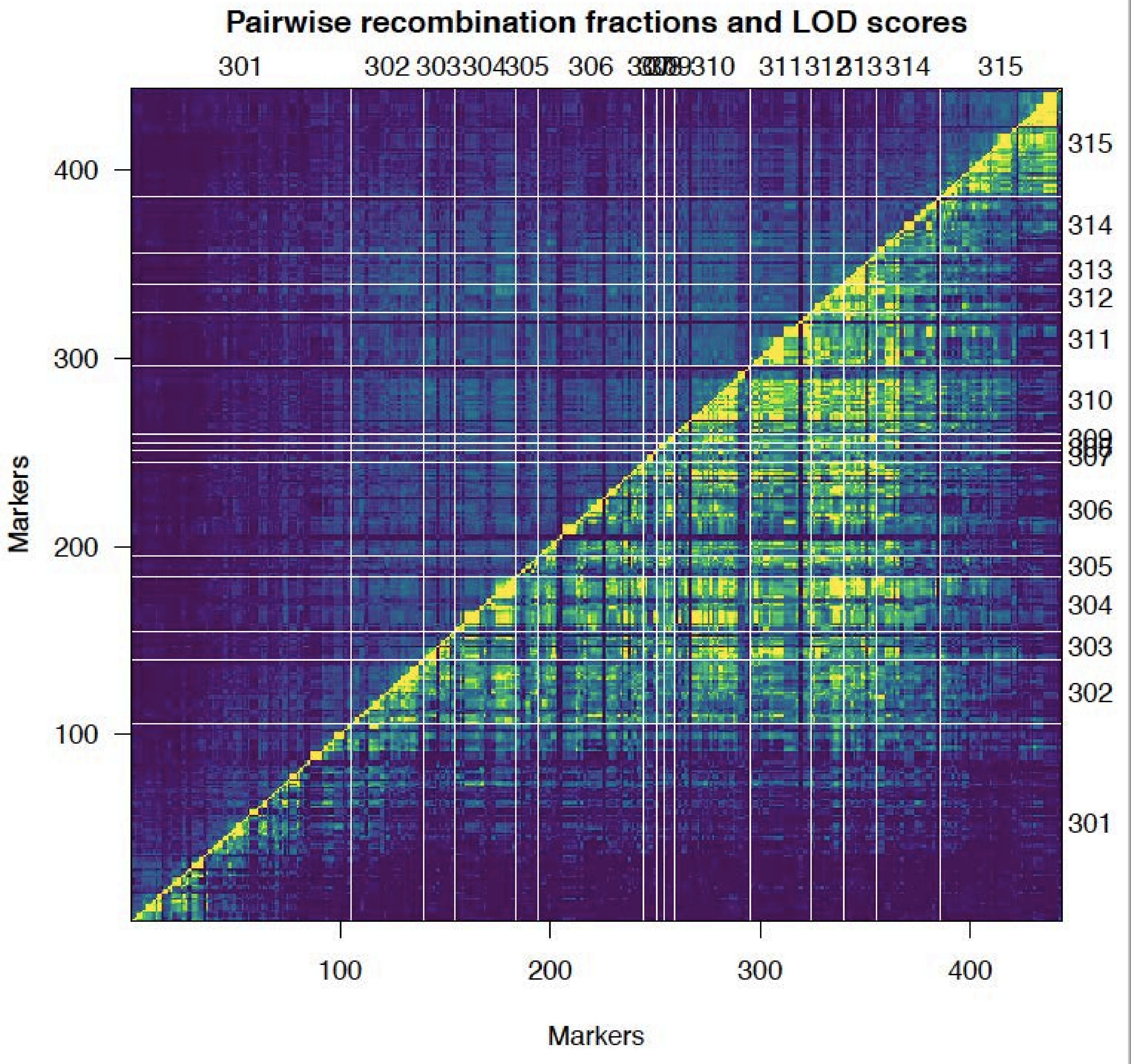

Pairwise recombination fractions and LOD scores
301
302
306
310
314
315
Markers
100
200
300
400
Markers

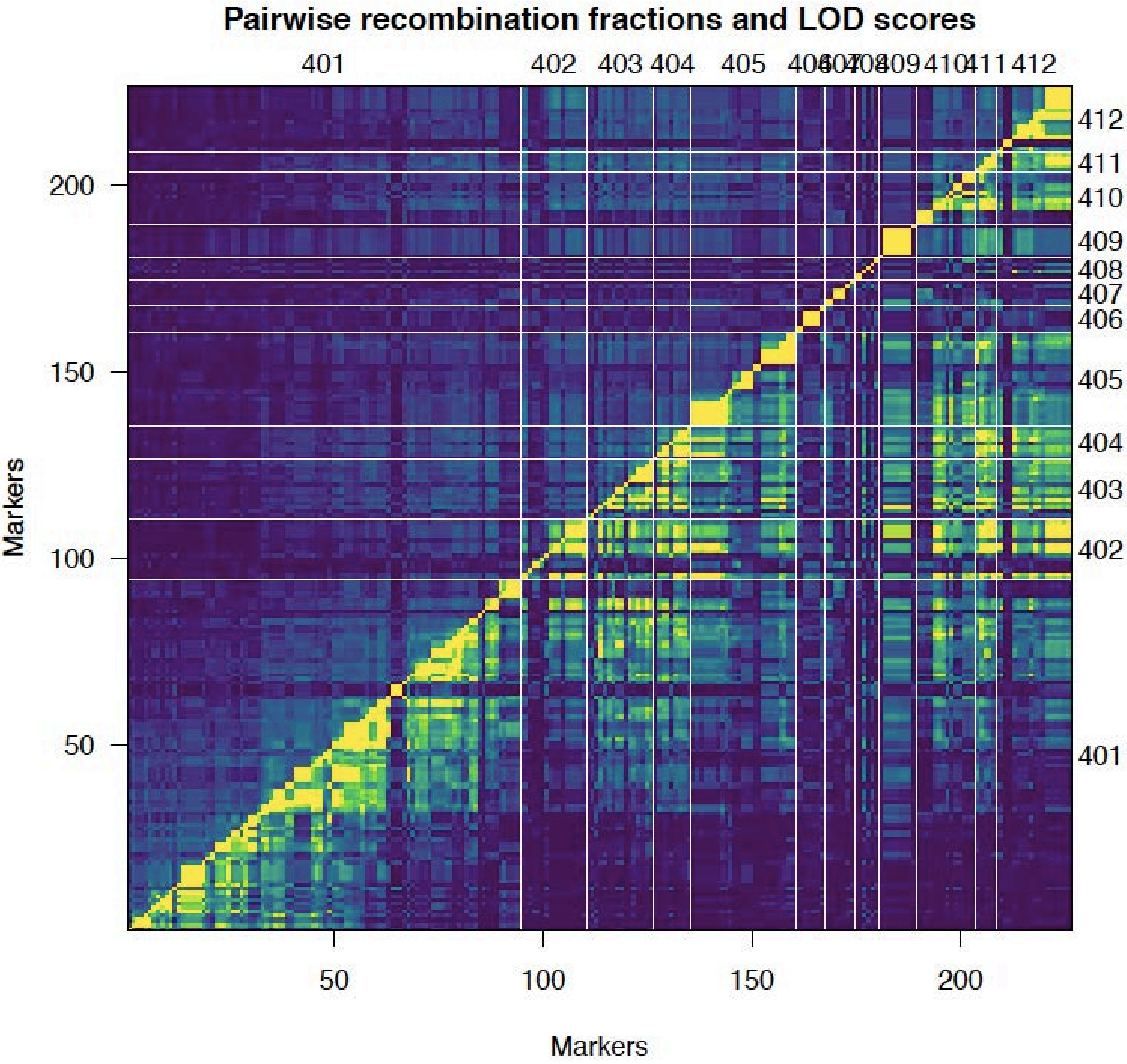

Pairwise recombination fractions and LOD scores
401
402
403
404
405
409
410
411
412
408
407
406
Markers
50
100
150
200
Markers

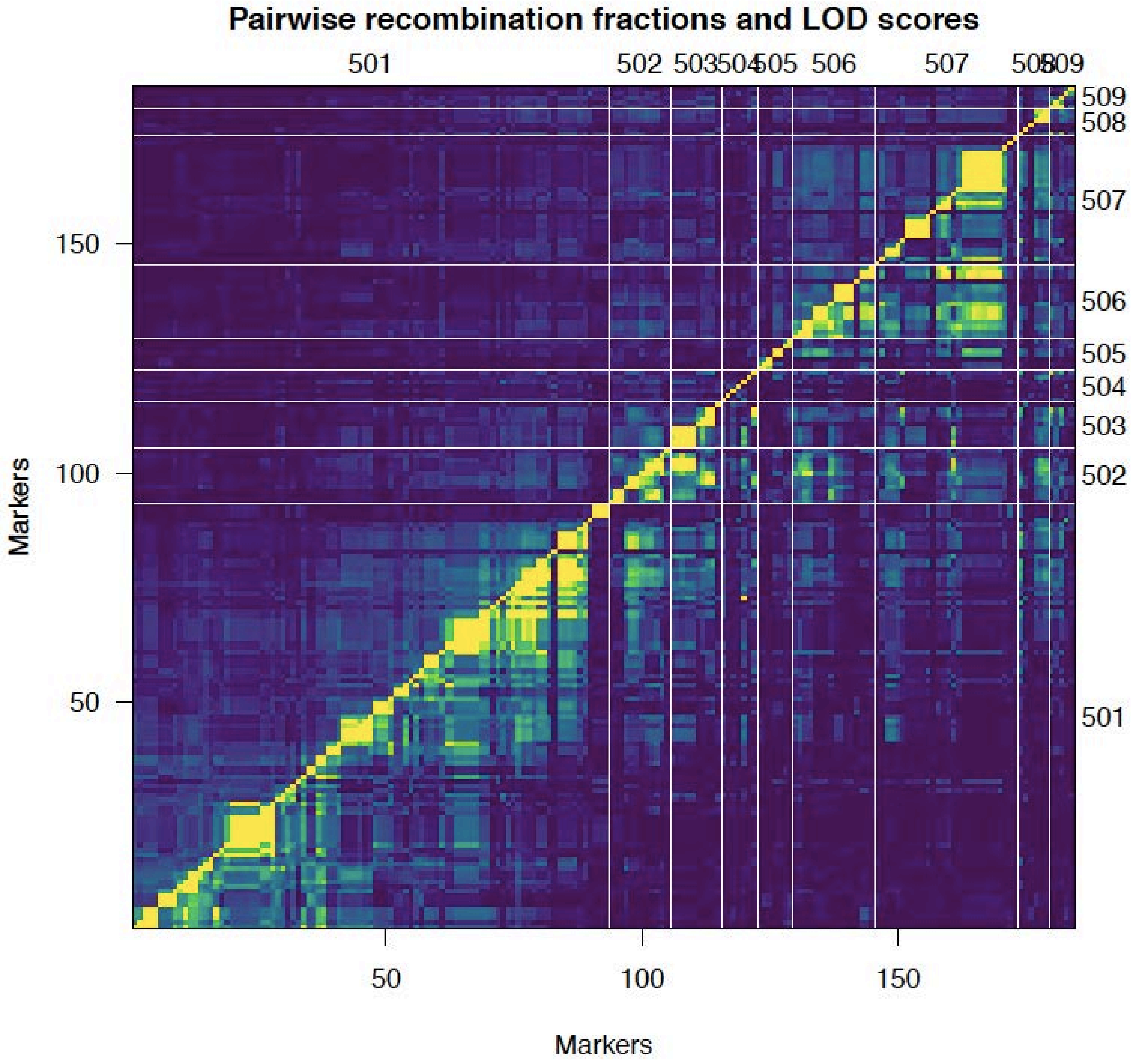

Pairwise recombination fractions and LOD scores
501
502
503
504
505
506
507
508
509
Markers
50
100
150
Markers
50
100
150

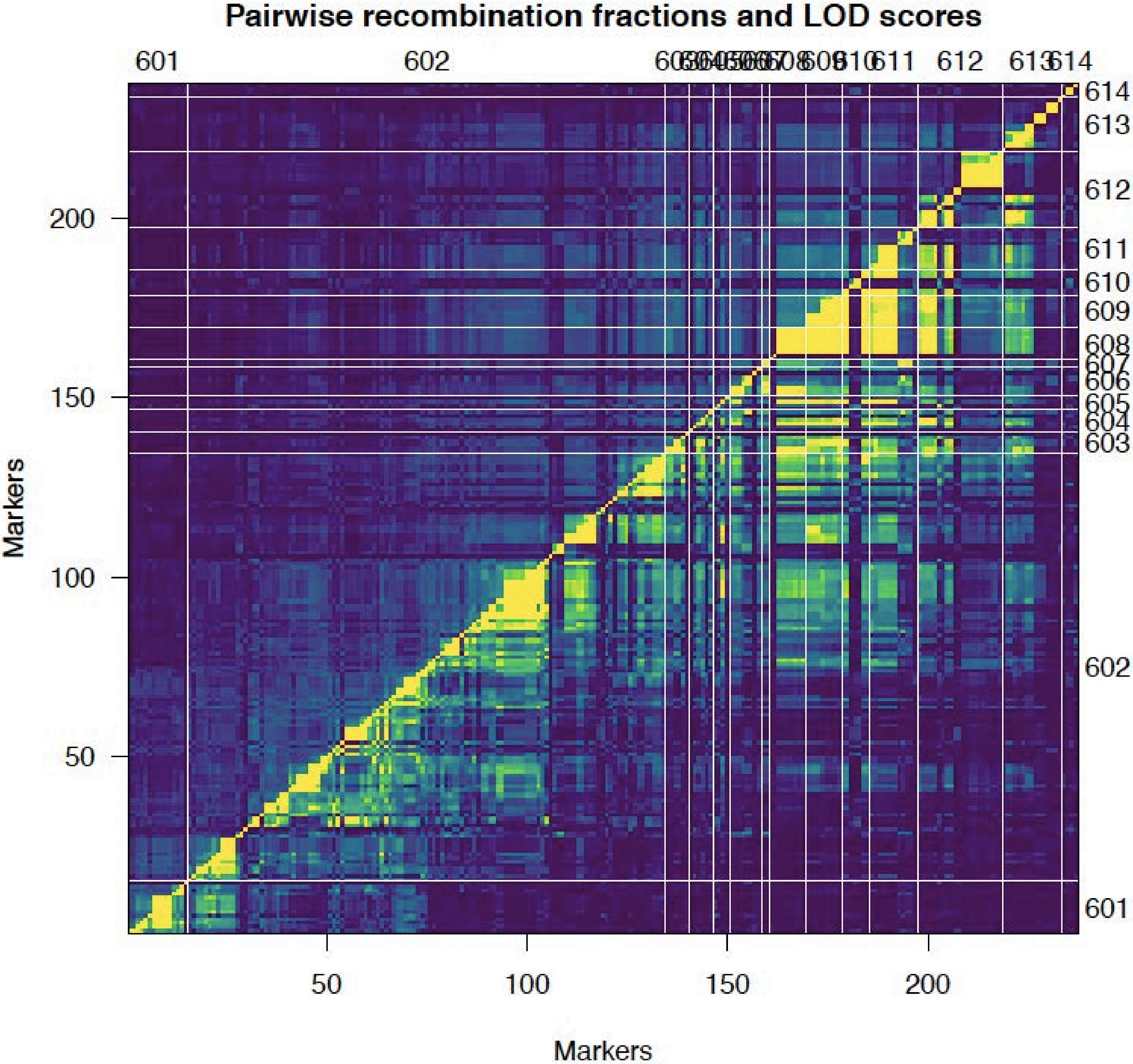

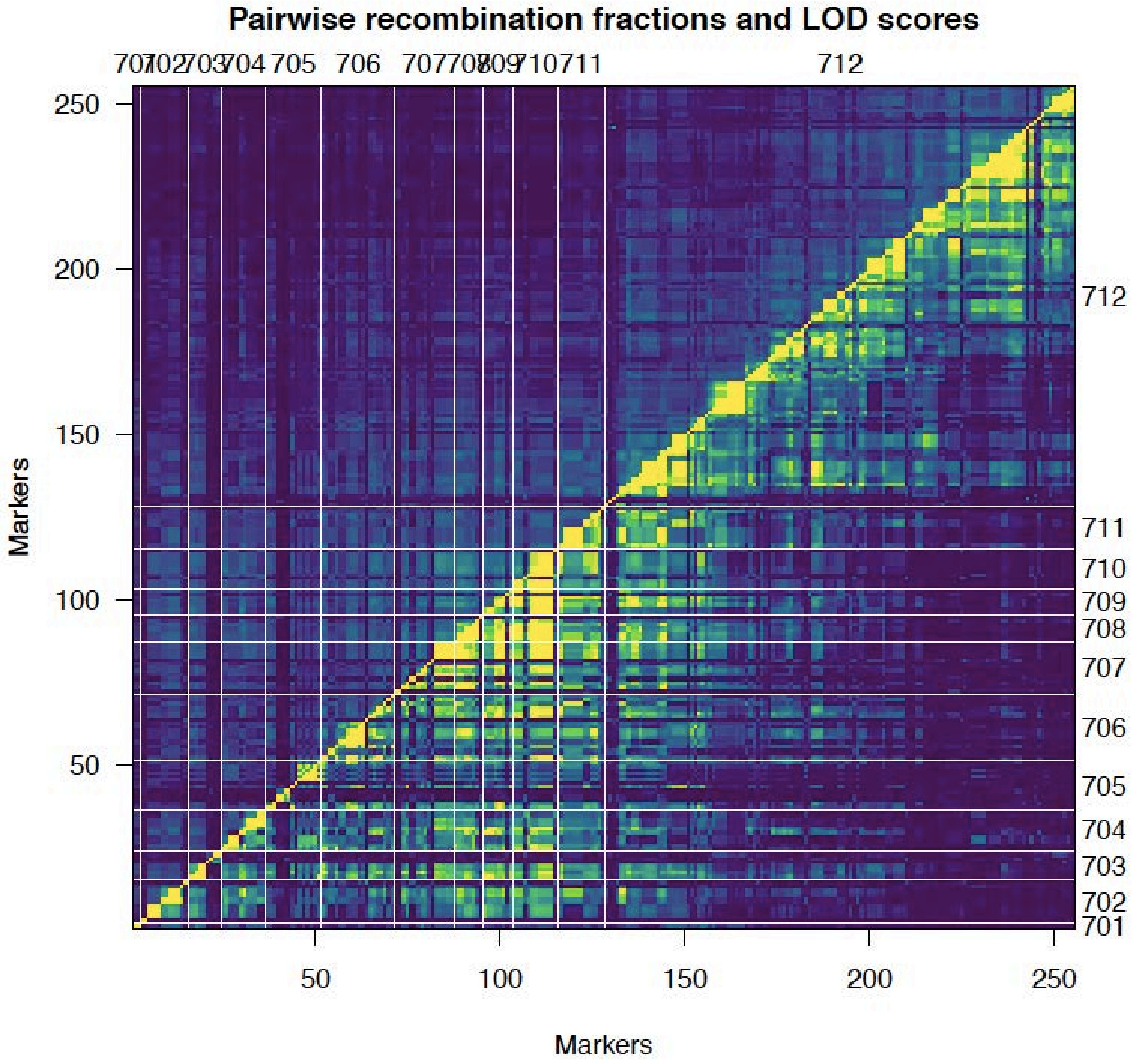
Pairwise recombination fractions and LOD scores
701
702
703
704
705
706
707
708
709
710
711
712
Markers
50
100
150
200
250
Markers
50
100
150
200
250
712
711
710
709
708
707
706
705
704
703
702
701

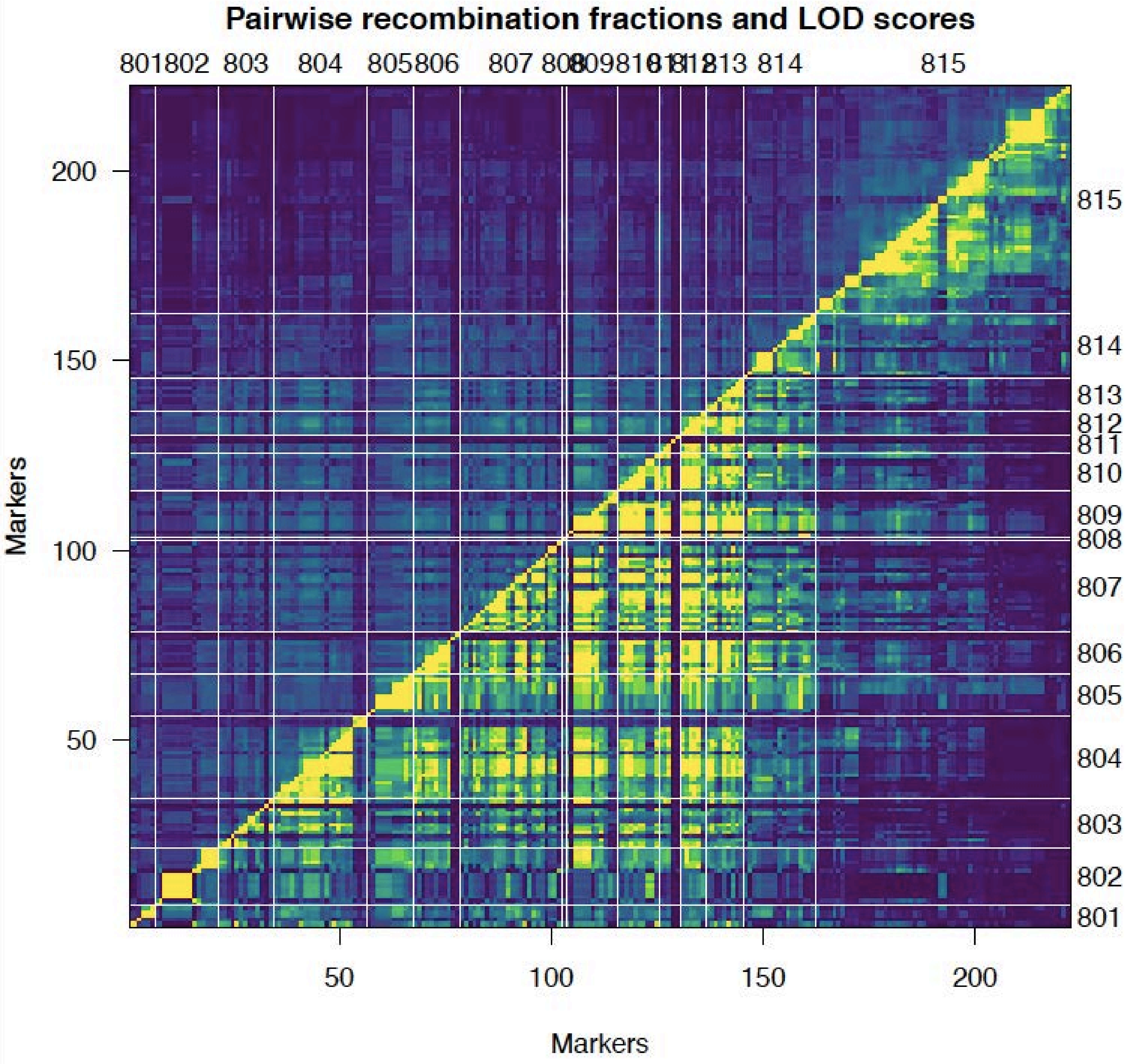

Pairwise recombination fractions and LOD scores
801
802
803
804
805
806
807
808
809
810
811
812
813
814
815
Markers
50
100
150
200
Markers

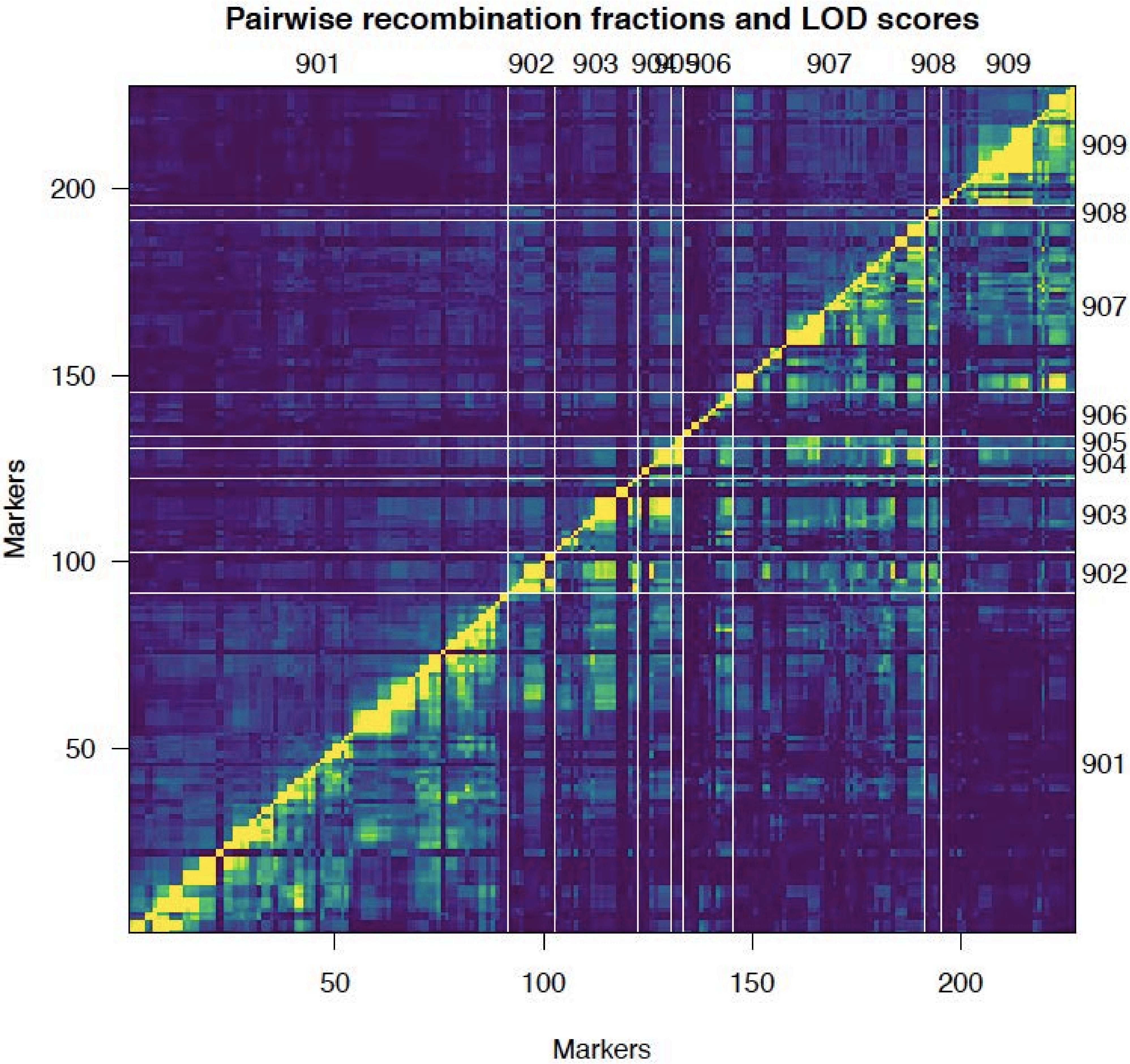

Pairwise recombination fractions and LOD scores
901
902
903
904
905
906
907
908
909
Markers
50
100
150
200
Markers
50
100
150
200
909
908
907
906
905
904
903
902
901

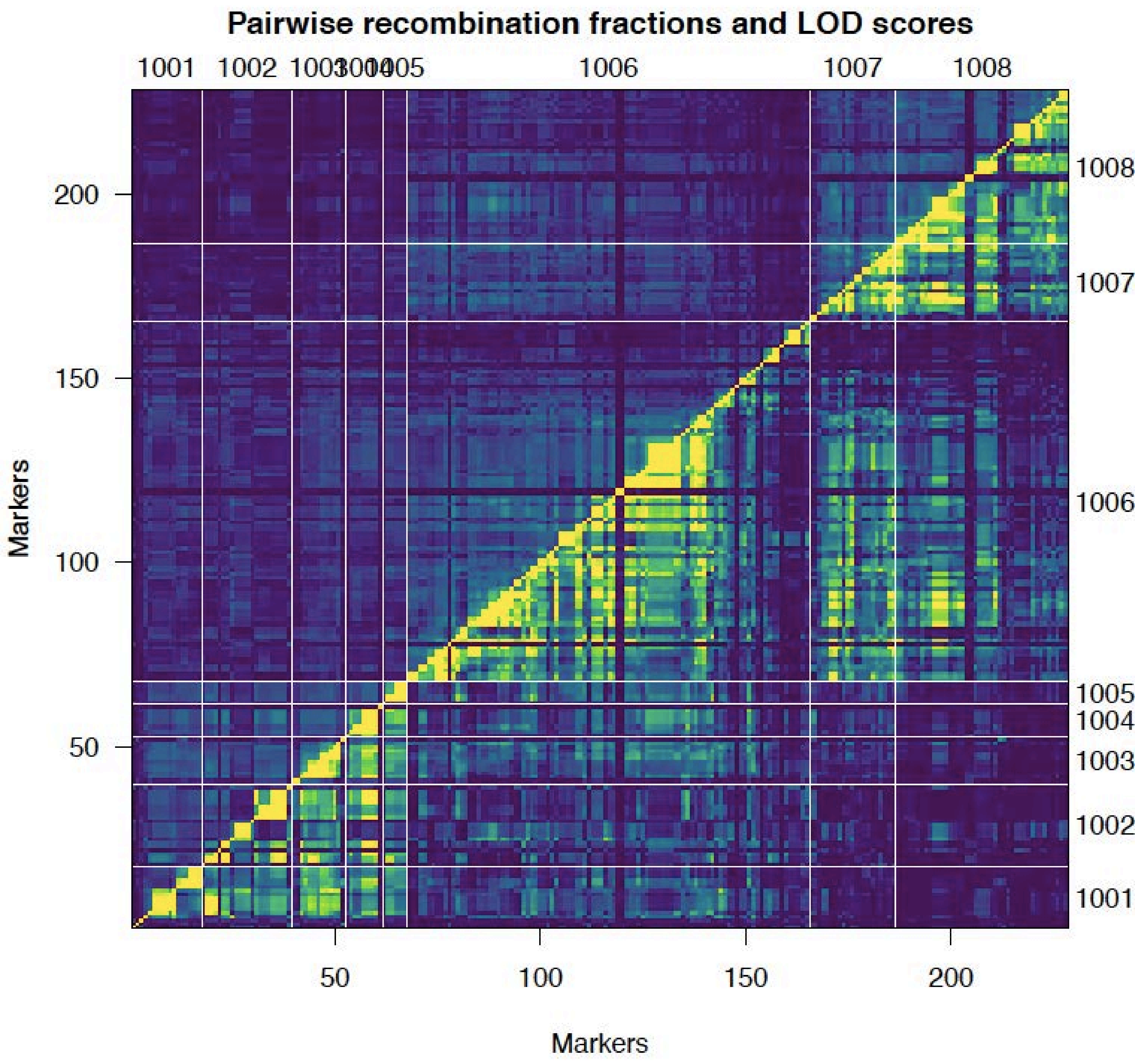

Pairwise recombination fractions and LOD scores
1001
1002
1003
1004
1005
1006
1007
1008
Markers
50
100
150
200
Markers
50
100
150
200
1008
1007
1006
1005
1004
1003
1002
1001

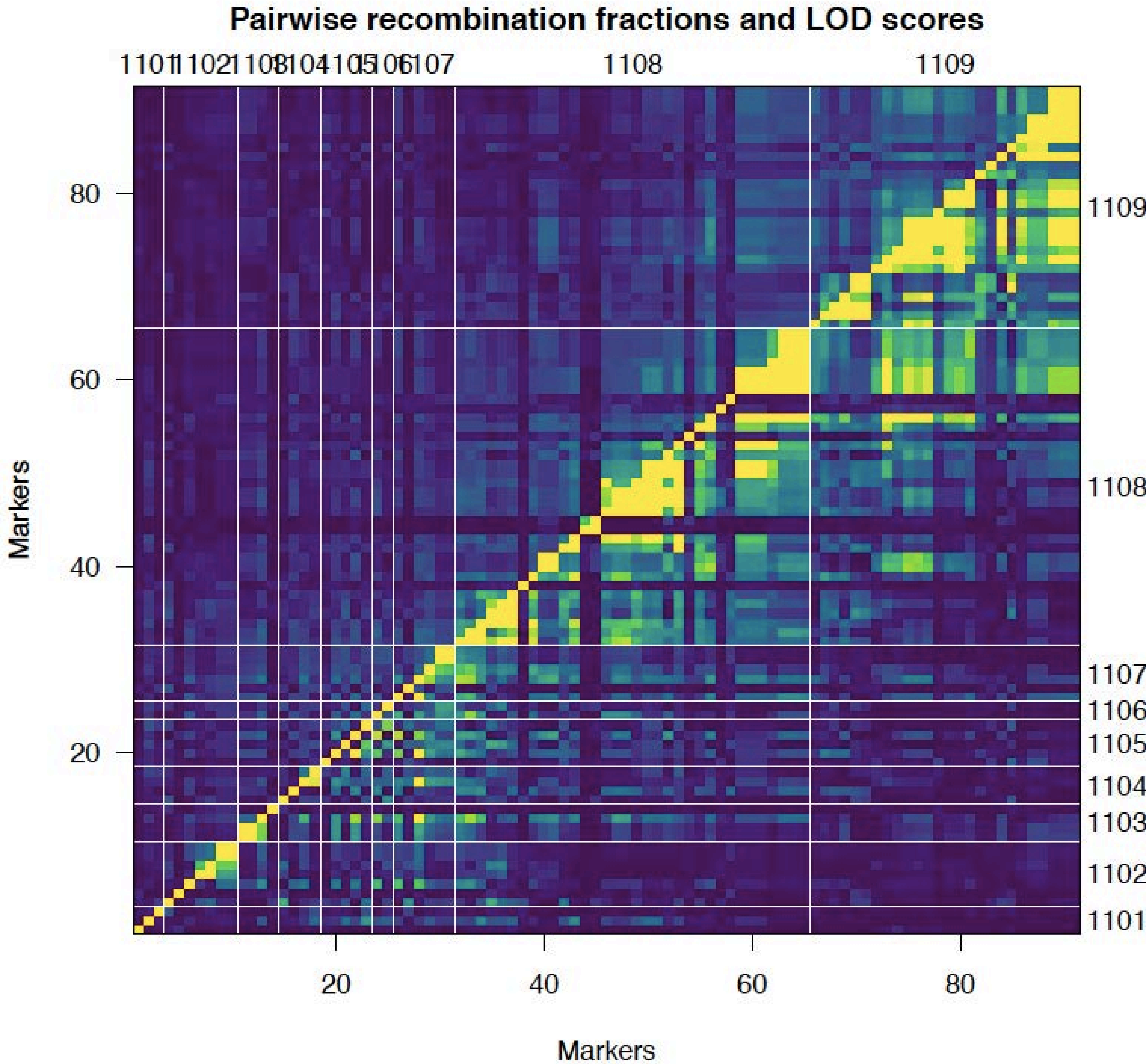

Pairwise recombination fractions and LOD scores
1101
1102
1103
1104
1105
1106
1107
1108
1109
Markers
20
40
60
80
Markers

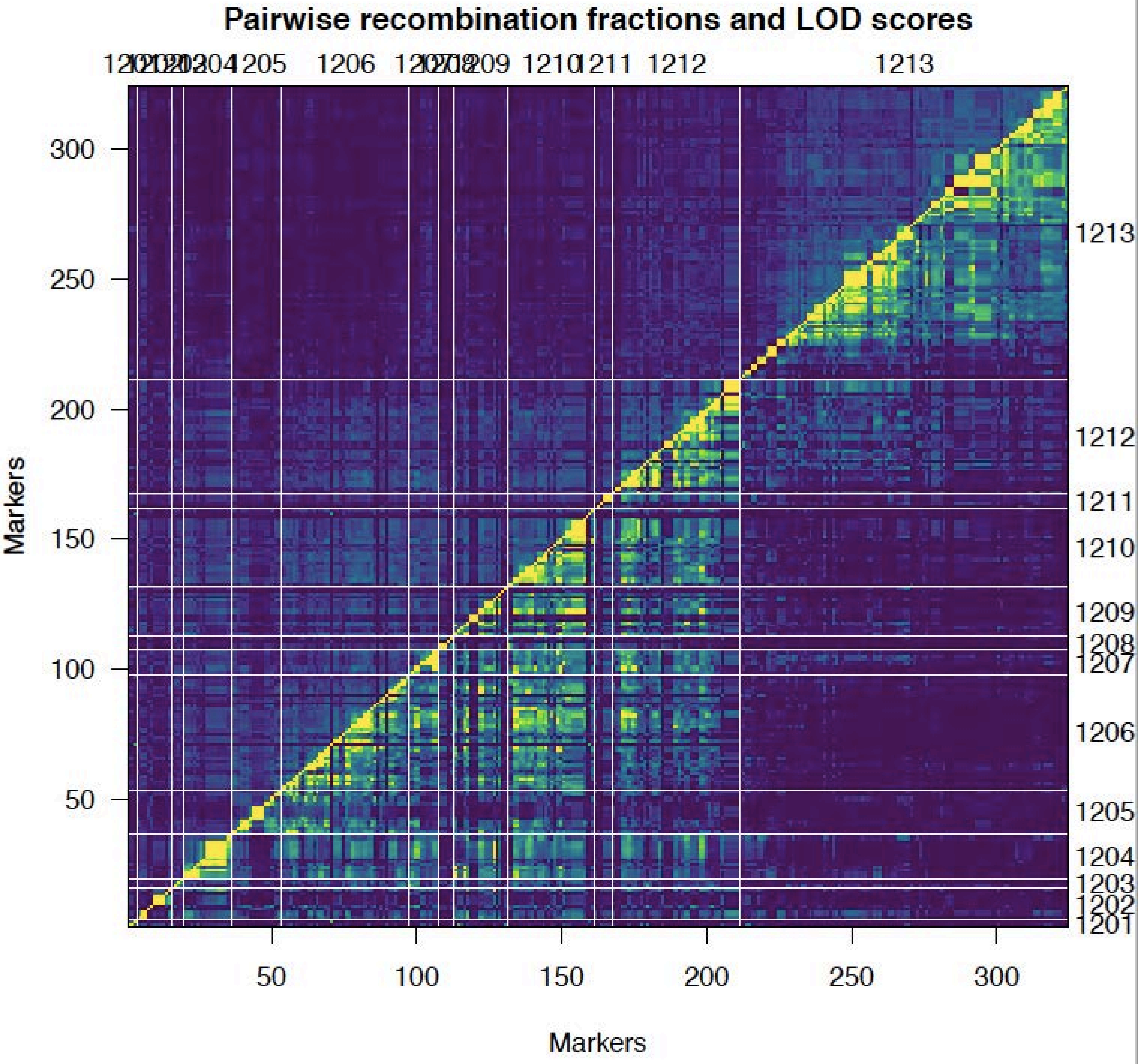
Pairwise recombination fractions and LOD scores
1205
1206
1209
1210
1211
1212
1213
300
250
200
150
100
50
Markers
1213
1212
1211
1210
1209
1208
1207
1206
1205
1204
1203
1202
1201
50
100
150
200
250
300
Markers

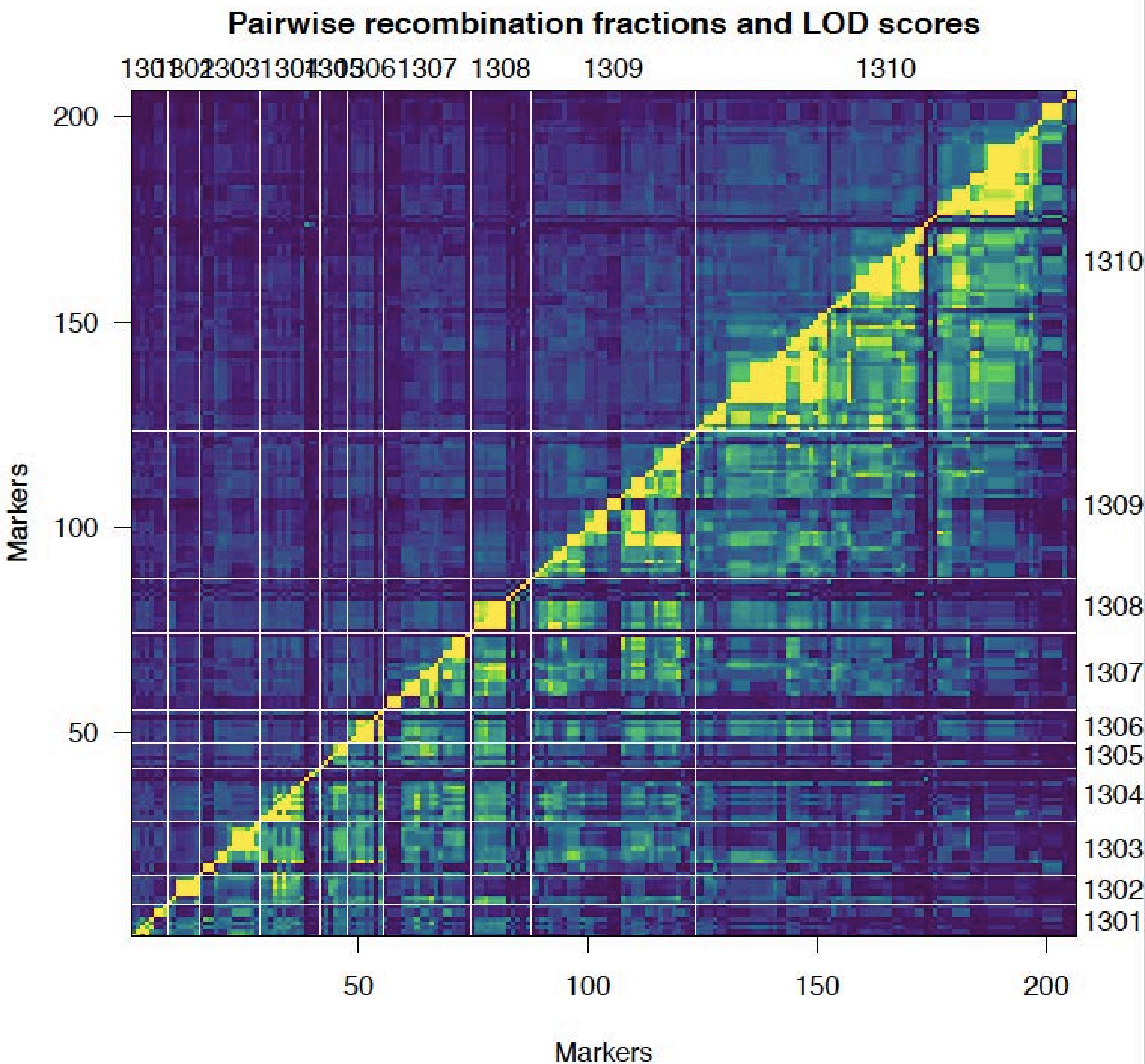
Pairwise recombination fractions and LOD scores
1307
1308
1309
1310
Markers
200
150
100
50
1310
1309
1308
1307
1306
1305
1304
1303
1302
1301
50
100
150
200
Markers

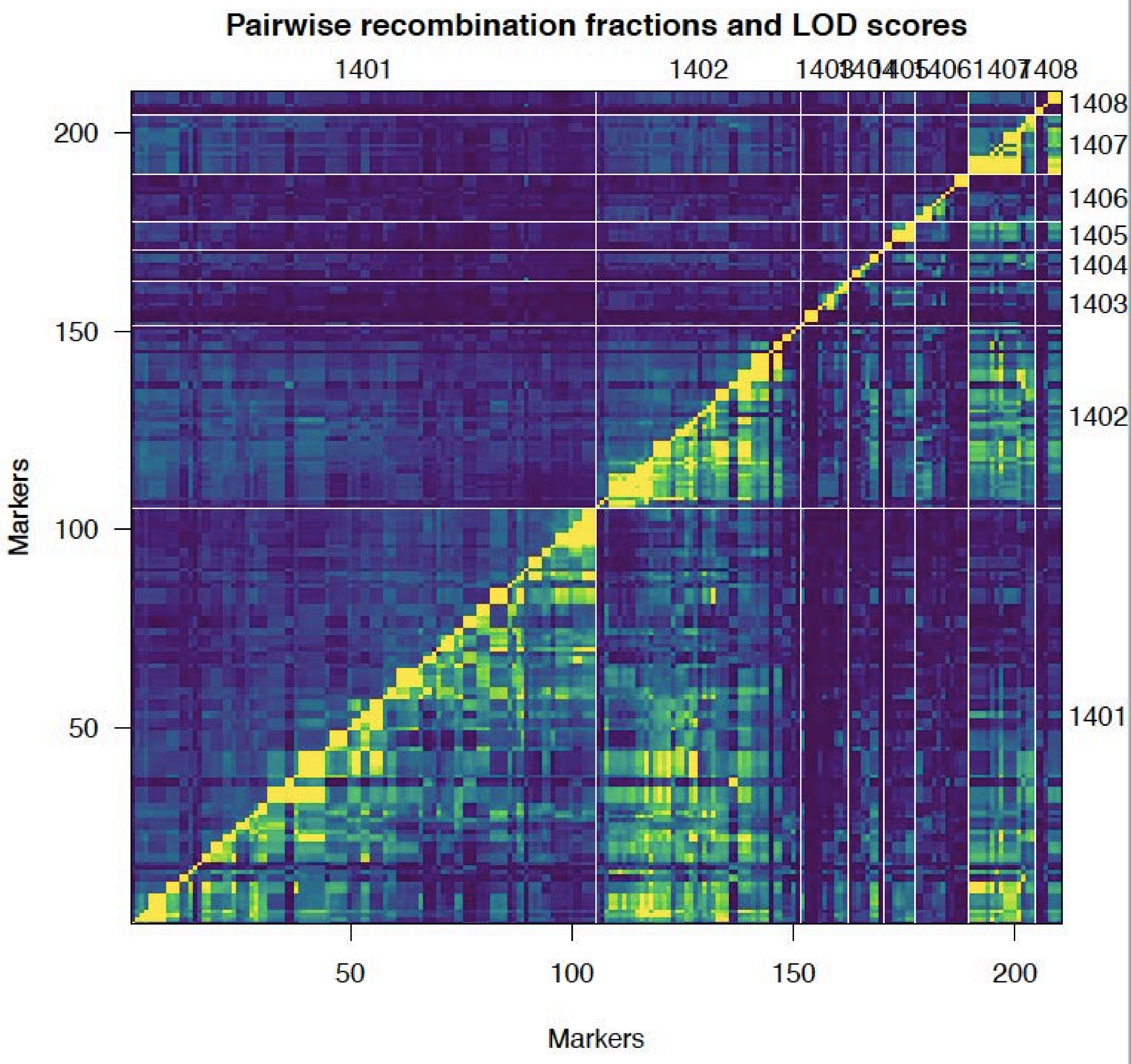
Pairwise recombination fractions and LOD scores
1401
1402
1403
1404
1405
1406
1407
1408
Markers
50
100
150
200
Markers
50
100
150
200

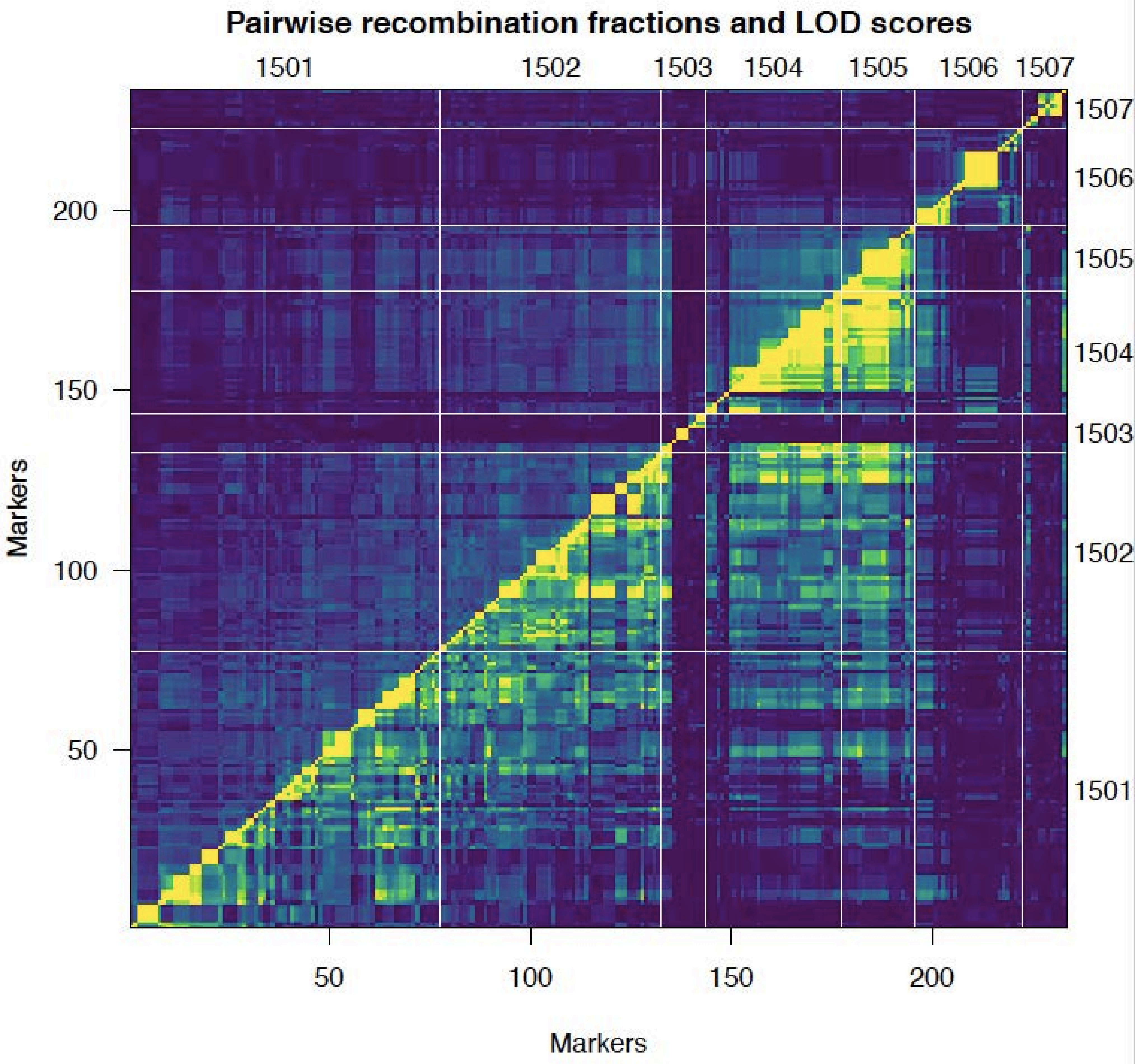
Pairwise recombination fractions and LOD scores
1501
1502
1503
1504
1505
1506
1507
Markers
50
100
150
200
Markers

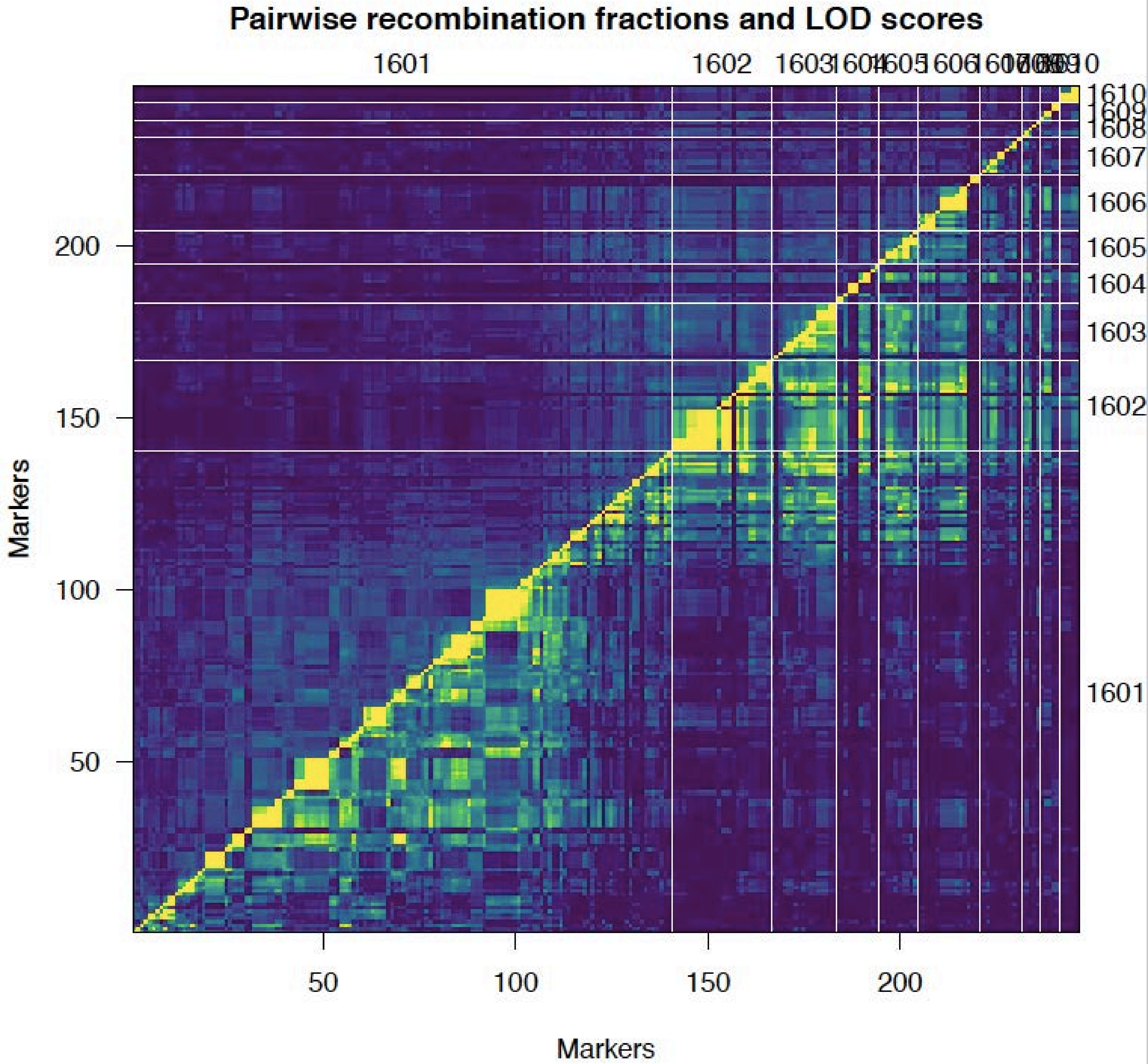

Pairwise recombination fractions and LOD scores
1601
1602
1603
1604
1605
1606
1610
1609
1608
1607
1606
1605
1604
1603
1602
1601
Markers
50
100
150
200
Markers
50
100
150
200

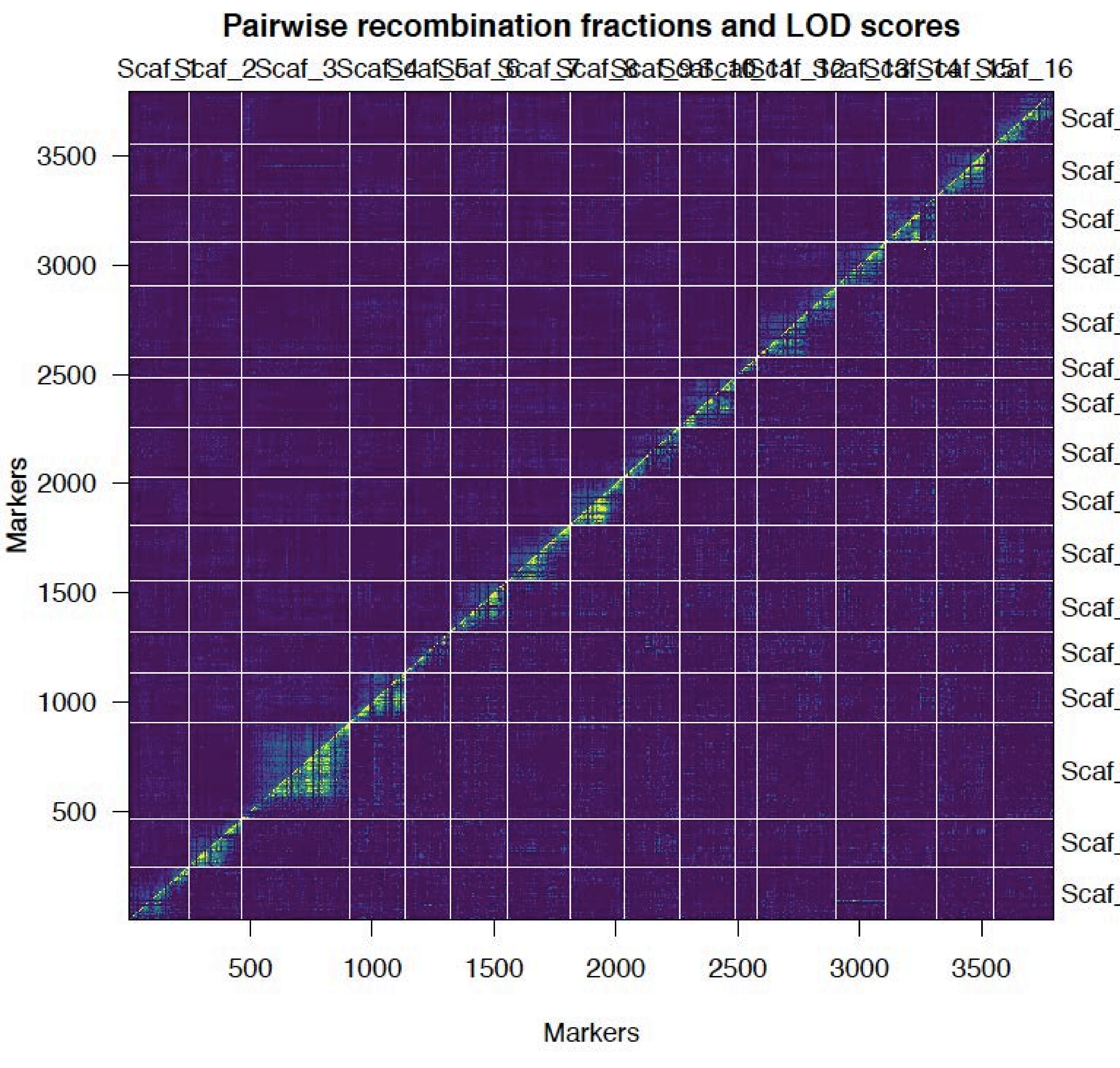
Pairwise recombination fractions and LOD scores
Markers
Markers
500
1000
1500
2000
2500
3000
3500
Scaf_16
