# Supplementary material for: Draft Genomes of Amaranthus tuberculatus, Amaranthus hybridus, and Amaranthus palmeri
Source: Genome Biol Evol. 2020 Aug 24;12(11):1988–93. doi: 10.1093/gbe/evaa177 (PMC7643611; doi:10.1093/gbe/evaa177)
Supplement: evaa177_Supplementary_Data [file evaa177_supplementary_data.zip › Supplemental captions.docx]

**Supplemental table and figure captions.**

**Table S1.** Comparison of contig order between POP1 and POP2 linkage maps. Shared contigs: present on both maps in the same order; unique contigs: present on only one map; misplaced contigs: present on both maps in a different order.

**Figure S1.** Distribution of depth across the *Amaranthus tuberculatus* (A) and *Amaranthus hybridus* (B) genome assemblies. Plot generated using the base package in R (R Core Team, 2018)

**Figure S2.** POP1 heatmaps indicating marker linkage (yellow = linked; blue = unlinked). Recombination fraction is shown in the upper left, LOD scores in the lower right. Maps 1 through 16: pairwise comparisons of markers within a linkage group by ordered contigs; Map 17: pairwise comparisons of markers across all linkage groups.
