# Supplementary material for: Draft Genomes of Amaranthus tuberculatus, Amaranthus hybridus, and Amaranthus palmeri
Source: Genome Biol Evol. 2020 Aug 24;12(11):1988–93. doi: 10.1093/gbe/evaa177 (PMC7643611; doi:10.1093/gbe/evaa177)
Supplement: evaa177_Supplementary_Data [file evaa177_supplementary_data.zip › Table S1.docx]

Table S1. Comparison of contig order between POP1 and POP2 linkage maps. Shared contigs: present on both maps in the same order; unique contigs: present on only one map; misplaced contigs: present on both maps in a different order.

| **Scaffold** | **Shared** | **POP1 unique** | **POP2 unique** | **Misplaced** |
| --- | --- | --- | --- | --- |
| 1 | 5 | 5 | 0 | 0 |
| 2 | 2 | 4 | 0 | 0 |
| 3 | 7 | 8 | 0 | 0 |
| 4 | 6 | 5 | 1 | 0 |
| 5 | 4 | 4 | 1 | 0 |
| 6 | 4 | 10 | 1 | 0 |
| 7 | 6 | 6 | 0 | 0 |
| 8 | 7 | 8 | 0 | 0 |
| 9 | 7 | 2 | 0 | 0 |
| 10 | 6 | 2 | 0 | 0 |
| 11 | 6 | 3 | 0 | 0 |
| 12 | 11 | 2 | 2 | 0 |
| 13 | 7 | 3 | 1 | 0 |
| 14 | 3 | 5 | 0 | 0 |
| 15 | 5 | 2 | 0 | 0 |
| 16 | 10 | 2 | 0 | 0 |
