# Supplementary figures and images for: Draft Genomes of Amaranthus tuberculatus, Amaranthus hybridus, and Amaranthus palmeri
Source: Genome Biol Evol. 2020 Aug 24;12(11):1988–93. doi: 10.1093/gbe/evaa177 (PMC7643611; doi:10.1093/gbe/evaa177)

### Depth Across *A. tuberculatus* Assembly

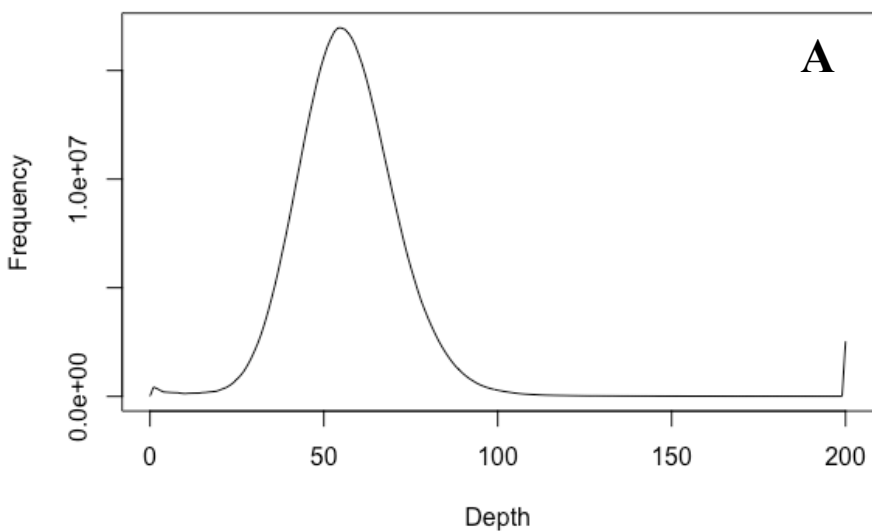

### Depth Across *A. hybridus* Assembly

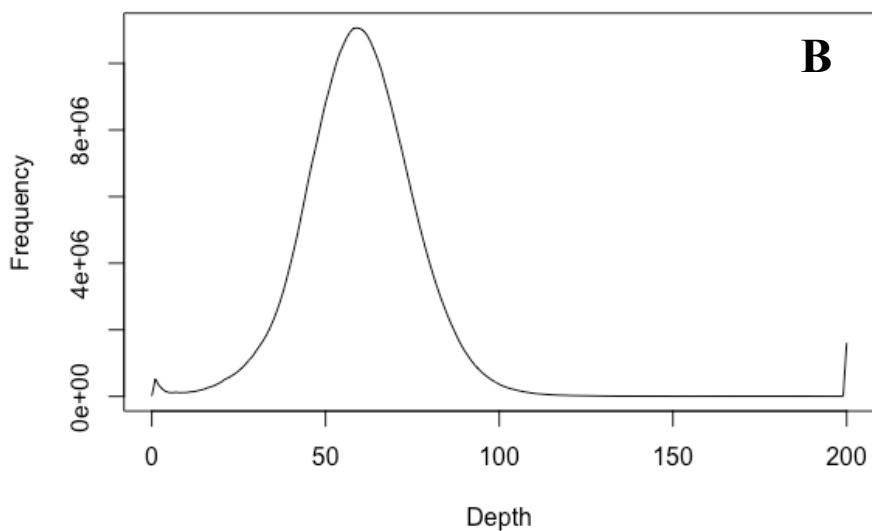

Supplement: evaa177_Supplementary_Data [file evaa177_supplementary_data.zip › Figure S1.pdf]
